# Supplementary material for: Video-evoked neuromarkers of visual function in age-related macular degeneration
Source: Front Hum Neurosci. 2025 May 1;19:1569282. doi: 10.3389/fnhum.2025.1569282 (PMC12078308; doi:10.3389/fnhum.2025.1569282)
Supplement: Supplementary file 1 [file Data_Sheet_1.pdf]

Supplementary Materials

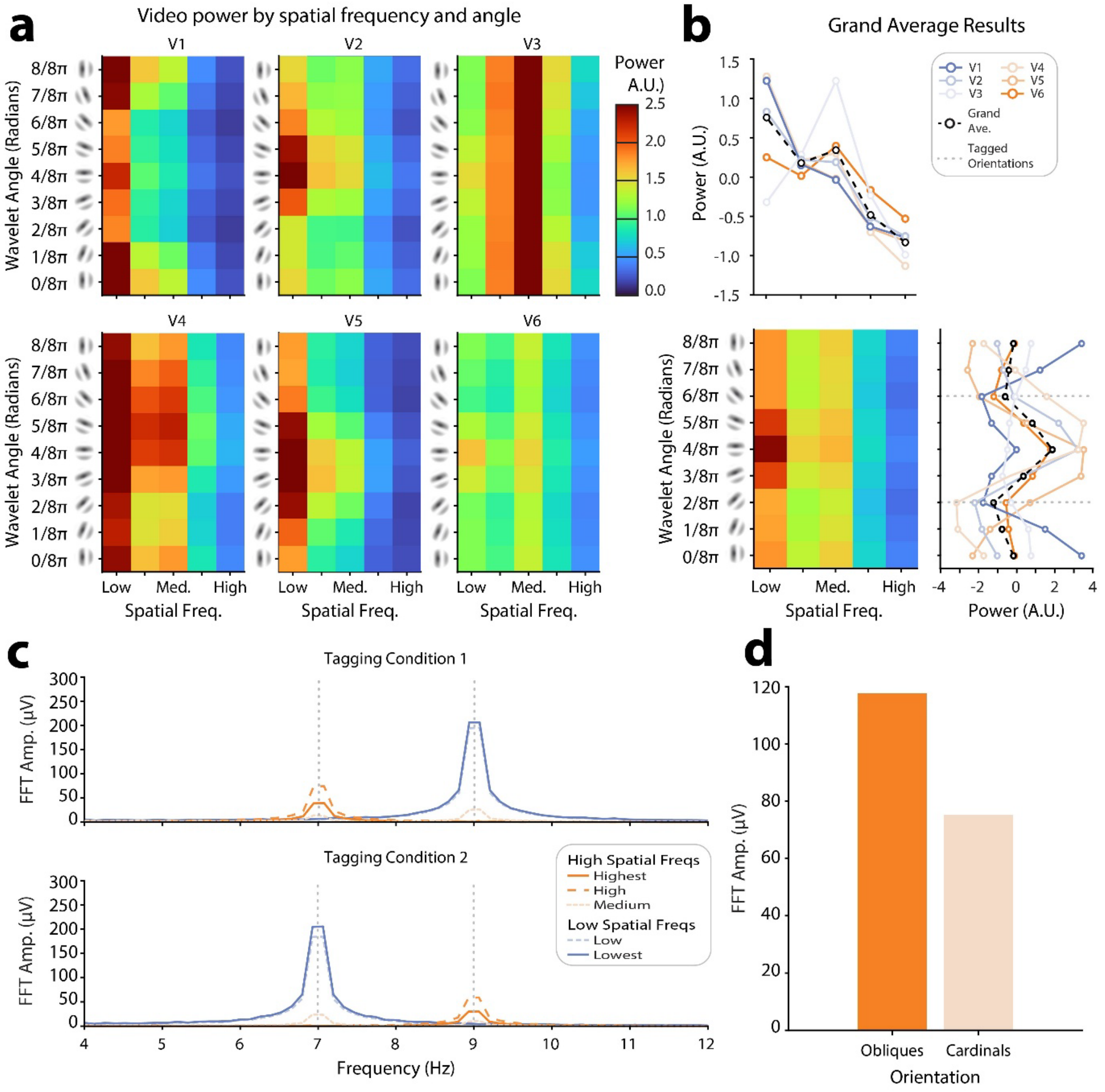

Supplementary Figure 1. Results of the 2D Steerable wavelet decomposition of the tagged video set. **a)** Videos were decomposed into 5 spatial frequency bands and 8 orientation bands. The average power across all video-frames for each video in the tagged set is shown for each spatial-frequency and orientation band. **b)** The average power for each spatial-frequency and orientation band across all videos shows that power peaked at horizontal angles of low spatial frequencies. The axes projecting from this image plot show the video-wise variations in power across orientation and spatial frequency. Note that in these projected axes, power has been baseline corrected for each video to allow for better visualisation of the within-video variance. **c)** To interrogate the fidelity of the frequency tagging procedure we computed the average power for oblique angles at each spatial frequency in each video-frame and subjected this power to an FFT. The power for each spatial frequency band is shown for each of the two tagged conditions (Condition 1: high SF- 7 Hz, low SF- 9 Hz | Condition 2: high SF: 9 Hz, low SF – 7Hz). Light-grey dashed lines show frequencies which were embedded into the videos. **d)** The same protocol was applied to cardinal angles. For each set of angles (oblique, cardinal), the power was taken at the tagged flicker frequency for each spatial frequency and for each tagging condition. This bar plot shows the average tagged power across these spatial frequencies and tagging conditions, for cardinal angles vs. obliques.
